# Allochrony is shaped by foraging niche segregation rather than adaptation to the windscape in long-ranging seabirds

**DOI:** 10.1186/s40462-024-00463-z

**Published:** 2024-04-02

**Authors:** Francesco Ventura, José Pedro Granadeiro, Paulo Catry, Carina Gjerdrum, Federico De Pascalis, Filipe Viveiros, Isamberto Silva, Dilia Menezes, Vítor H Paiva, Mónica C Silva

**Affiliations:** 1 Biology Department, Woods Hole Oceanographic Institution, Woods Hole, MA USA; 2 CESAM, Departamento de Biologia Animal, Faculdade de Ciências, Universidade de Lisboa, Campo Grande, 1749-016 Lisboa, Portugal; 3 MARE - Marine and Environmental Sciences Centre / ARNET - Aquatic Research Network, Ispa - Instituto Universitário, Rua Jardim do Tabaco 34, 1149-041 Lisboa, Portugal; 4 Canadian Wildlife Service, Environment and Climate Change Canada, B2Y 2N6 Dartmouth, NS Canada; 5 Area Avifauna Migratrice, Istituto Superiore per la Protezione e la Ricerca Ambientale (ISPRA), Ozzano dell’Emilia, Italy; 6 Parque Natural da Madeira, Quinta do Bom Sucesso, Caminho do Meio, 9050-251 Funchal, Madeira, Portugal; 7 MARE - Marine and Environmental Sciences Centre / ARNET - Aquatic Research Network, Department of Life Sciences, University of Coimbra, Calçada Martim de Freitas, 3000-456 Coimbra, Portugal; 8 Centre for Ecology, Evolution and Environmental Changes (cE3c), Departamento de Biologia Animal, Faculdade de Ciências, Universidade de Lisboa, Campo Grande, 1749-016 Lisboa, Portugal

**Keywords:** Allochrony, Ecological segregation, Flight behaviour, Foraging niche, *Pterodroma*, Seabird, Stable isotope, Wind

## Abstract

**Background:**

Ecological segregation allows populations to reduce competition and coexist in sympatry. Using as model organisms two closely related gadfly petrels endemic to the Madeira archipelago and breeding with a two month allochrony, we investigated how movement and foraging preferences shape ecological segregation in sympatric species. We tested the hypothesis that the breeding allochrony is underpinned by foraging niche segregation. Additionally, we investigated whether our data supported the hypothesis that allochrony is driven by species-specific adaptations to different windscapes.

**Methods:**

We present contemporaneous tracking and stable isotopes datasets for Zino’s (*Pterodroma madeira*) and Desertas (*Pterodroma deserta*) petrels. We quantified the year-round distribution of the petrels, characterised their isotopic niches and quantified their habitat preferences using machine learning (boosted regression trees). Hidden-Markov-models were used to investigate the effect of wind on the central-place movement speed, and a simulation framework was developed to investigate whether each species breeds at times when the windscape is most favourable to sustain their trips.

**Results:**

Despite substantial spatial overlap throughout the year, the petrels exhibited diverging isotopic niches and habitat preferences during breeding. Both species used a vast pelagic region in the North Atlantic, but targeted two different mesopelagic ecoregions and showed a preference for habitats mostly differing in sea surface temperature values. Based on our simulation framework, we found that both species would perform trips of similar speed during the other species’ breeding season.

**Conclusions:**

The different breeding schedules between the species are underpinned by differences in foraging habitat preferences and adaptation to the local environment, rather than to the windscape. Nevertheless, the larger Desertas petrels exploited significantly windier conditions, potentially unsustainable for the smaller Zino’s petrels. Furthermore, due to larger mass and likely higher fasting endurance, Desertas petrels engaged in central-place-foraging movements that covered more ground and lasted longer than those of Zino’s petrels. Ultimately, patterns of ecological segregation in sympatric seabirds are shaped by a complex interplay between foraging and movement ecology, where morphology, foraging trip regulation and fasting endurance have an important– yet poorly understood– role.

**Supplementary Information:**

The online version contains supplementary material available at 10.1186/s40462-024-00463-z.

## Introduction

According to the principle of competitive exclusion, ecologically similar sympatric populations cannot coexist unless they evolve characters specialised for different niches and partition their use of available resources [1]. The evolution of phenotypic traits stemming from selection to reduce competition (“character displacement”) is a fundamental mechanism shaping the structure of communities and promoting divergence in the foraging niches of competing species [2–5]. Strong past competition and consequent divergence in phenotypic traits can be fixed in time through generations, resulting in present patterns of segregation between populations, even in those with small numbers compared to their historical abundance (‘the ghost of competition past’ [6]). Such segregation can involve one or multiple foraging niche dimensions: for instance, foragers may use different areas, at different times, or exploit different habitats to consume different prey, as documented empirically in several sympatric marine predators including fish [7], marine mammals [8–10] and seabirds [11, 12].

Phenotypic differences in morphology and body size are often indicative of resource partitioning, as they set physiological limits to a species’ foraging ability and strategy [13, 14]. For example, structural differences in beaks influence prey selection [15]. Body size and associated oxygen storage capacity limit the attainable diving depth and duration in air-breathing vertebrates [16, 17]. In some avian taxa, including seabirds belonging to the order *Procellariiformes* (i.e., albatrosses, petrels and shearwaters), flight morphology and body size (e.g., wing loading and aspect ratio) determines flight costs and selection/avoidance for specific wind conditions [18, 19].

Many seabird species are apex predators playing a key role in the world oceans, congregating in breeding colonies often comprising of thousands to millions of individuals [20]. Seabirds are useful model organisms to investigate density-dependent competition and ecological segregation. Over the past decades, several studies documented segregation in seabird spatial distribution at sea [15], allochrony in breeding phenology [21] and dietary specializations [22]. For many pelagic seabirds, locomotor efficiency is key to optimal foraging, particularly so during the breeding season, when they are central-place foragers [23]. Yet, despite the central-place constraint, some seabirds travel hundreds to thousands of kilometres to forage over immense ocean areas in search of ephemeral and heterogeneously distributed prey patches [24, 25]. Such hypermobility is underpinned by a flight behaviour known as “dynamic soaring”, with which seabirds extract aerodynamic kinetic energy from the wind to minimize flight costs [26, 27].

Gadfly petrels (genus *Pterodroma*) are the largest group of procellariiform seabirds, comprising some of the rarest and most threatened seabird species, but their ecology remains poorly understood. In this work, we focus on two sympatric gadfly petrels, the Zino’s petrel (*Pterodroma madeira*) and the Desertas petrel (P. *deserta*). The two species breed exclusively in the Madeiran archipelago in close geographical proximity (approximately 40 km apart), in the central mountain massif of Madeira (Zino’s petrel) and on Bugio island (Desertas petrel), with extremely small population sizes, estimated at ca. 160 (Zino’s petrel) and 200 (Desertas petrel) breeding pairs [28]. Only in recent years Zino’s and Desertas petrels were classified as two distinct species [29], and their evolutionary divergence is thought to be relatively recent (ca. 40,000 years) [30]. As in other gadfly petrels [31–34], both Zino’s and Desertas petrels are exceptionally wide ranging and highly mobile throughout their annual cycle [35]. They are both solitary foragers, and although little is known about their diet, they exploit mesopelagic trophic resources ([36] and own unpublished data), opportunistically caught over deep, pelagic waters [25, 37].

The two study species largely overlap in their distribution [35, 37] and are morphologically very similar. The main differences between them include their bill morphology [29], body size (Zino’s petrel weighs approximately 200 g and Desertas petrel 300 g [38]) and wingspan (Zino’s petrel 800–843 mm, Desertas petrel 860–940 mm [36]). Based on allometric relationships derived empirically on *Procellariiformes* [39], the wing area of the Zino’s and Desertas petrel should be 460 cm^2^ and 584 cm^2^, respectively, leading to a wing loading of 0.44 g/cm^2^ and 0.51 g/cm^2^. Nevertheless, the observed difference in wingspan between species (∼10%) should theoretically lead to a 33% increase in mass and not to the observed 50% increase. The wing-loading of the Desertas petrel should therefore not only be higher than that of Zino’s petrel, but also higher than expected based on simple allometry. Such differences in flight morphology may in turn determine selectivity for different wind conditions by the two species, with higher wing loadings being advantageous for flight in stronger winds [18]. Furthermore, there is a marked temporal segregation in the breeding schedule of the two species, with the breeding season of Zino’s petrels (April to October) starting and ending 2 months earlier than that of Desertas petrels (June to December) [35]. All these characteristics make Zino’s and Desertas petrels a uniquely valuable case study to investigate the links between phenology, foraging ecology, windscape and locomotor efficiency.

In this work, we quantify patterns of segregation in the foraging niches of these two petrels focussing on their use of space and habitat preferences, inferred from both stable isotopes and tracking data. The latter represent the most comprehensive spatial datasets for the two species, including some of the few available GPS tracking data for these gadfly petrels, and the first for the Zino’s petrel. The overarching aim of this research is to understand the drivers of breeding allochrony between the two petrels. Our main hypothesis is that:

**(*Hp*)** The breeding allochrony is underpinned by species-specific adaptations to exploit different foraging niches. To test this hypothesis, we investigate whether, by breeding two months apart, petrels partition their use of space and foraging habitat.

Wind plays a key role in shaping the foraging ecology of dynamic soaring seabirds by modulating their locomotory efficiency [19, 24, 40]. Given their long-ranging movements, this is particularly true for gadfly petrels, which should experience strong selective pressure to optimise wind use and achieve efficient flight. Therefore, we also test the support for the hypothesis that the breeding allochrony is driven by adaptations to exploit different windscapes, with each species breeding at times when the windscape is most favourable to undertake their long central place foraging movements. Under this hypothesis, we predict that the ground speed (which, in this study, is the metric with which we evaluate the petrels’ flight performance) attained by each species during their own breeding season is higher than that achievable during the breeding season of the other species.

## Methods

### Data collection

Combined geolocator-immersion loggers (“GLS”, Intigeo C65, Migrate Technology Ltd, total weight of 1 g) were leg-mounted on breeding Zino’s petrels (*n* = 8 tagged birds, ∼2.5% of the total population) and Desertas petrels (*n* = 11 tagged birds, ∼2.8% of the total population). The tags recorded time, light intensity and saltwater immersion, i.e. periods in which the GLS– and therefore the tagged petrels– were dry (in flight) or wet (on the water). GLS deployments lasted between July 2019– June 2020 (for Zino’s petrel) and September 2019– August 2020 (for Desertas petrel). The GLS light intensity data were processed using the probabilistic algorithm from the R package probGLS [41] to estimate the most likely movement trajectory for each individual (supplementary information).

GPS loggers (nanoFix, Pathtrack Ltd, weight of 3.4 g) were deployed during incubation of the breeding seasons of 2018 and 2019 (Zino’s petrel); and of 2015, 2016, 2017 and 2019 (Desertas petrel). Loggers were taped to the four central tail feathers. The weight of loggers and tape combined was less than 3% of the average body mass (supplementary information). All tracking datasets were linearly interpolated (at 1 or 2 h resolution, see below) using the R package adehabitatLT [42] to impute missing data and obtain tracks regularly spaced in time. The extent of interpolation was minimal (with less than 2% of the points being imputed). The petrels undertook both long foraging trips and shorter foraging movements closer to the colony. Using k-mean clustering, we assigned each track to a “long” and “short” category based on the distance from colony and duration (supplementary information). As we were unsure of the function of the short tracks, which represented less than 13% of the total recorded time spent at-sea by the tracked animals (supplementary information) and are perhaps not primarily linked to foraging, only the long tracks were retained for the analysis. The resulting GPS tracking dataset for the analysis comprised: 12 tracks from 9 individuals at a 1 h temporal resolution (Zino’s petrel); and 22 trips from 19 individuals at 1 h resolution plus 21 trips from 16 individuals at 2 h resolution (Desertas petrel).

Wind grids at 10 m altitude above the ocean were downloaded at a spatio-temporal resolution of 0.25° and 1 h from the ECMWF ERA-5 database (https://cds.climate.copernicus.eu/cdsapp). For each track point, we extracted the following variables: wind direction (expressed in degrees); wind intensity (ms^− 1^); wind direction relative to bird movement direction (“Δangle”) and tail wind component (“TWC”) calculated as in [25]. The TWC quantifies the wind speed component in the direction of the bird movement. The Δangle variable is bounded between 0° (representing tail winds aligned with the bird’s direction of movement) to 180° (representing head winds blowing against the direction of movement).

### Year-round spatial overlap and flight activity

For each species, we separated the GLS data into six 2-month seasonal windows (starting from September to October and ending with July-August). The datasets were collected simultaneously, with the exception of the data from July and August (2019 and 2020 were considered for Zino’s and Desertas petrel, respectively). For each species and each seasonal window, we used the R package adehabitatHR [43] to compute Utilization Distributions (UDs). We used a smoothing parameter *h* = 2.25° (approximately equal to 250 km); quantified UDs for each individual on 0.25° resolution grids, rescaling the values of the grids so that their sum added up to 1; we extracted the mean UD (across individuals) for each species and seasonal window. Finally, we quantified the proportion of spatial overlap (i.e., the proportion of area of a species’ UD overlapping with that of the other species) in the space use of the two species across the yearly cycle (i.e., for each seasonal window). To test whether the observed 2-month asynchrony in their breeding phenology contributes to reduce the yearly spatial overlap, we synchronised the Zino’s petrel breeding cycle with that of Desertas petrel by adding two months to their real GLS data time-stamp, and carried out the same overlap analysis described above. To describe the year-round activity patterns of the petrels, we considered the wet/dry periods recorded by the GLS (supplementary information).

### Isotopic niche

Stable isotope ratios are biogeochemical tracers used to define predator distributions and their trophic interactions. Variation in the nitrogen isotopic ratio (δ^15^N) is used as an indicator of the trophic position of a consumer, whereas the carbon isotopic signature (δ^13^C) provides spatial information on its distribution (e.g. [15, 31]).. However, recent studies on isoscapes (i.e., spatial distribution models of stable isotope ratios) showed that, over large spatial scales, baseline isotopic signatures are not homogeneous [44]. This is the case for the North Atlantic, where different oceanic regions are characterised by different baseline isotopic signatures [44]. Spatio-temporal variability in baseline δ^15^N may ultimately obscure signals on the trophic position of the consumers [45]. In this context, here we investigate differences in the isotopic niche of the two species as indicators of the usage of different habitats, i.e. of water masses with different biogeochemical properties, associated with divergences in combined spatial distribution and diet.

We analysed carbon and nitrogen stable isotope values in blood samples of incubating birds during the 2018 and 2019 breeding seasons (*n* = 24 Zino’s petrels; *n* = 25 Desertas petrels). Stable isotope analyses of whole blood were carried out using continuous flow isotope mass spectrometry on a Sercon Hydra 20–22 (Sercon, UK) spectrometer, coupled to a EuroEA (EuroVector, Italy) elemental analyser. Isotope ratios were expressed adopting the δ notation in parts per thousand (‰) relative to V-PDB scale (δ^13^C) and AIR scale (δ^15^N). Internal laboratory standards assessment indicated that the measurement error was ≤ 0.1‰ for δ^13^C and δ^15^N (supplementary information).

### Behavioural classification

Discrete-time hidden-Markov-models (HMMs) were fit to the GPS central-place-foraging tracks using the package momentuHMM [46] in R. For each species, HMMs were used to classify the behavioural states of the birds along the tracks based on the distance travelled (step length) and the change of movement direction (turning angle) observed at each movement step. The most likely sequence of behavioural states was inferred using the Viterbi algorithm built in the momentuHMM package. In the models, we assumed that, along the tracks, petrels were in one of the following behavioural states: “transit”, in which birds are moving at high speed at a persistent heading; or “search”, in which the underlying behaviour is to engage in food search upon reaching a foraging patch [46]. Further details of the model can be found in the supplementary information.

Two sets of HMMs were formulated. In the first set, the objective was to obtain the locations chosen as “search” points by the two species, to then investigate the foraging habitat preferences during incubation (see Sect. 2.5 below). For these HMMs, we considered all Zino’s petrel tracks and all Desertas petrel tracks (i.e., collected at 1 h and at 2 h temporal resolution), and the latter were resampled at 2 h resolution to minimise the amount of interpolated points, increasing biological realism. In the second HMM set, the objective was to compare the effect of wind on the petrel ground speed considering the locations classified as “transit” (see Sect. 2.6 below). To minimize potential biases, for this analysis we only considered GPS tracks collected at the same temporal resolution for both species (i.e., 1 h).

### Habitat model

To investigate the habitat preferences of the two species, we built environmental niche models using boosted regression tree (BRT) machine-learning algorithms [47]. Here, we only considered the track locations classified as “search” by the HMMs above. The search points were assigned a value of “1” (i.e., presence). For each presence, 3 at-sea random points were drawn and were coded as “0”. The latter represent the “pseudo-absences”, i.e. those locations that were available but not used by the birds. The pseudo-absences were drawn from within an area with spatial extent equal to the maximum longitudinal and latitudinal range of the tracks of both species. For each species, we extracted the pseudo-absences from a land-free circular buffer centred in the colony with radius equal to 110% of the maximum distance from colony attained by the focal species. In the BRTs (hereafter referred to as “*habitat models*”), our binomial response variable (i.e., the set of presences and pseudo-absences) was modelled as a function of physiographic, oceanographic, biological and distance-related explanatory variables, which we hypothesised to affect the petrel probability of presence (supplementary information). The variables tested were: distance from the colony; distance from seamounts (considering seamounts at depths between 0 and 500 m below the surface); wind speed; bathymetric depth and slope; sea surface temperature (“SST”) and sea surface temperature gradient; sea surface height above sea level; eddy kinetic energy; chlorophyll A concentration; density ocean mixed layer thickness; mass content of epipelagic, migrant upper mesopelagic and highly migrant lower mesopelagic micronekton in sea water (supplementary information).

For each species, the *habitat models* quantified the relative importance of each explanatory variable in shaping the habitat suitability for foraging petrels. The relative importance values were expressed as percentage and their sum was equal to 100%. *Habitat models* were fit to the data with the bernoulli loss function, using the gbm.step function from the dismo package [48] in R, adopting the hyper-parametrization described in the supplementary information. The predictive performance of the *habitat models* was evaluated using K-fold cross-validation metrics [47] (supplementary information).

### Wind model and track simulation

We used GAMMs (hereafter referred to as “*wind models*”) from the R package mgcv [49] to investigate the effect of wind on the ground speed (i.e. the step length) of birds when they were in the “transit” state. We assumed that, when in this state, the relationship between the ground speed of birds and wind would not be biased by the other activities performed by birds when searching for food. The gamma distribution was used to model the response variable (ground speed). The explanatory variables Δangle and wind intensity were included as cubic regression splines with shrinkage; their tensor product interaction was also tested in the model. To account for temporal autocorrelation, the *wind models* were formulated using the auto-regressive AR1 correlation structure, applied to each individual track (random effect) at regular time-steps.

Based on the species-specific relationship between ground speed and wind estimated by the respective *wind model*, we used a simulation framework (supplementary information) to ask:

- Does the windscape explain species-specific differences in breeding distribution, or could they achieve equivalent flight performance by carrying out the other species’ tracks? To address this question, we evaluated the simulated flight performance of a focal species (e.g., Zino’s petrel) undertaking the tracks of the other species (e.g., those realised by Desertas petrel) during its own (Zino’s petrel) breeding season.
- Does the windscape explain species-specific differences in breeding schedules, or could they achieve equivalent flight performance during the breeding season of the other species? To test this, we evaluated the simulated flight performance of a focal species (e.g., Zino’s petrel) undertaking its observed tracks during the breeding season of the other species (e.g., the Desertas petrel breeding season).

**Fig. 1:**
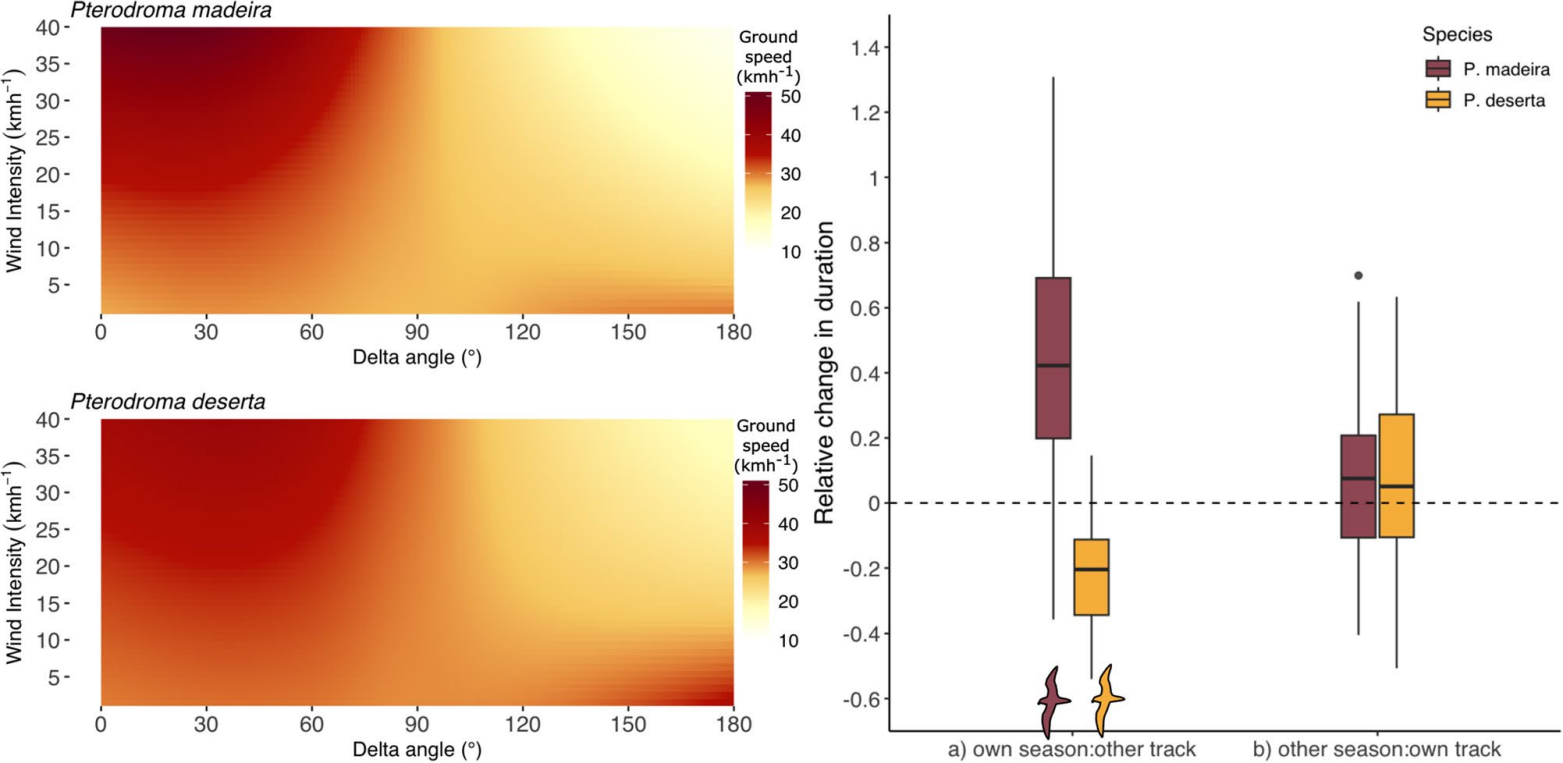
– Left panel. Heatmap depicting the results of the *wind model*, showing the effect of wind Δangle and wind intensity on the ground speed of the two species. Right panel. Boxplot showing the relative difference in temporal duration required to complete the real (“own”) and simulated (“other”) tracks performed by *Pterodroma madeira* (in red) and *P. deserta* (in gold), during their observed breeding season (“own season”) and during that of the other species (“other season”). For each species, the relative difference in duration was calculated using as reference the average duration of the real data (i.e., “own” tracks carried out during their “own” season). The box represents the interquartile range and the solid line shows the median duration. The petrel silhouettes highlight significant differences in track duration (see results in main text)

In this simulation, flight performance was exclusively evaluated in terms of the ground speed and resulting temporal duration necessary to complete the tracks under different simulated scenarios. For the reasons outlined above, we only considered the movement steps classified as “transit” by the HMMs. For each species, the temporal duration of the simulated tracks was compared to that of the real (observed) tracks and the % change in duration (and ground speed) was calculated (Fig. 1).

## Results

### Year-round spatial overlap and flight activity

**Fig. 2:**
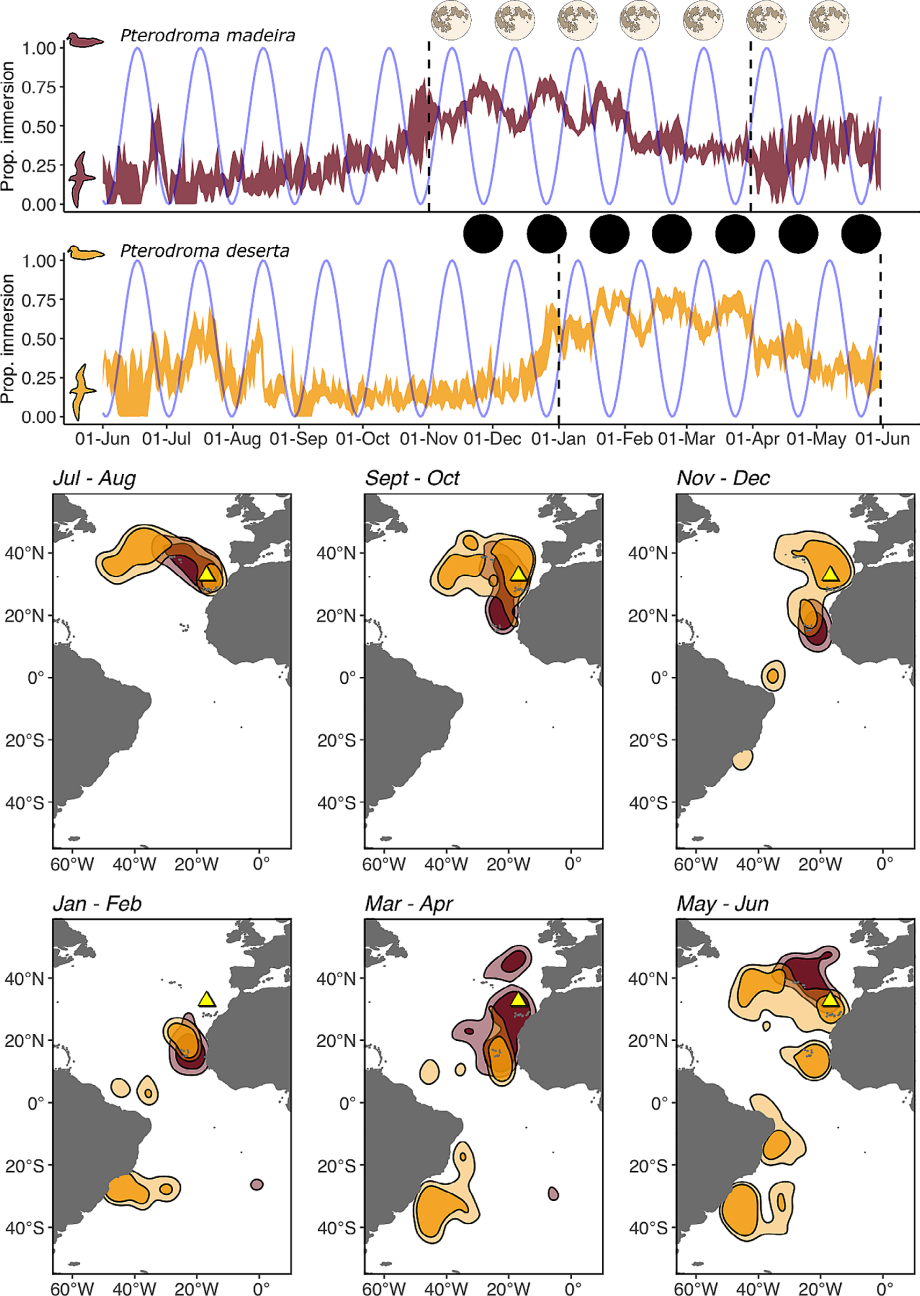
– Top two rows: the year-round activity of Zino’s petrels (*Pterodroma madeira*) in red and Desertas petrels (*P. deserta*) in gold, estimated based on the daily proportion of saltwater immersion recorded by the GLS. The overlaid solid blue line shows the proportion of moon illumination for the respective dates (supplementary information), highlighting days of full moon and new moon (shown as a dark circle). For each species, the non-breeding season occurs between the vertical dashed black lines. Bottom two rows: the yearly overlap in the spatio-temporal distribution of *Pterodroma madeira* (in red) and *P. deserta* (in gold). For each panel, the shapes represent the 50% (opaque) and 75% (transparent) UD contours estimated based on the GLS data. The two breeding colonies are depicted by the yellow triangles. Given their geographical proximity (distance ∼ 40 km), the two triangles overlap

The two species exhibited a varying degree of overlap in their 50% UD contour (defined hereafter as the core spatial distribution) throughout the year (Fig. 2). Overall, 29% of the Zino’s petrel core distribution overlapped with that of the Desertas petrel. The overlap was highest in January-February (47%) and September-October (35%).. Considering the Desertas petrel core distribution, the spatial overlap with the Zino’s petrel was, on average, equal to 17%, peaking in January-February (21%), March-April (29%) and September-October (21%). If petrels were to breed at the same time, their spatial overlap would increase (on average, 52% of the Zino’s petrel and 26% of the Desertas petrel core distribution overlapped with that of the other species).

Both Zino’s and Desertas petrels spent a larger portion of their daily activity in flight during the breeding season compared to the non-breeding season. After excluding the days in which the petrels were in the nest, the average daily proportion of time spent on the water during breeding was 0.30 (Zino’s petrel) and 0.32 (Desertas petrel). During non-breeding, they spent less time flying and more time on the water, with a daily saltwater immersion estimated at 0.51 and 0.52 for Zino’s and Desertas petrel, respectively. During the non-breeding season of both species, the petrels’ flight activity peaked during nights when moon illumination was at its highest (Fig. 2).

**Fig. 3:**
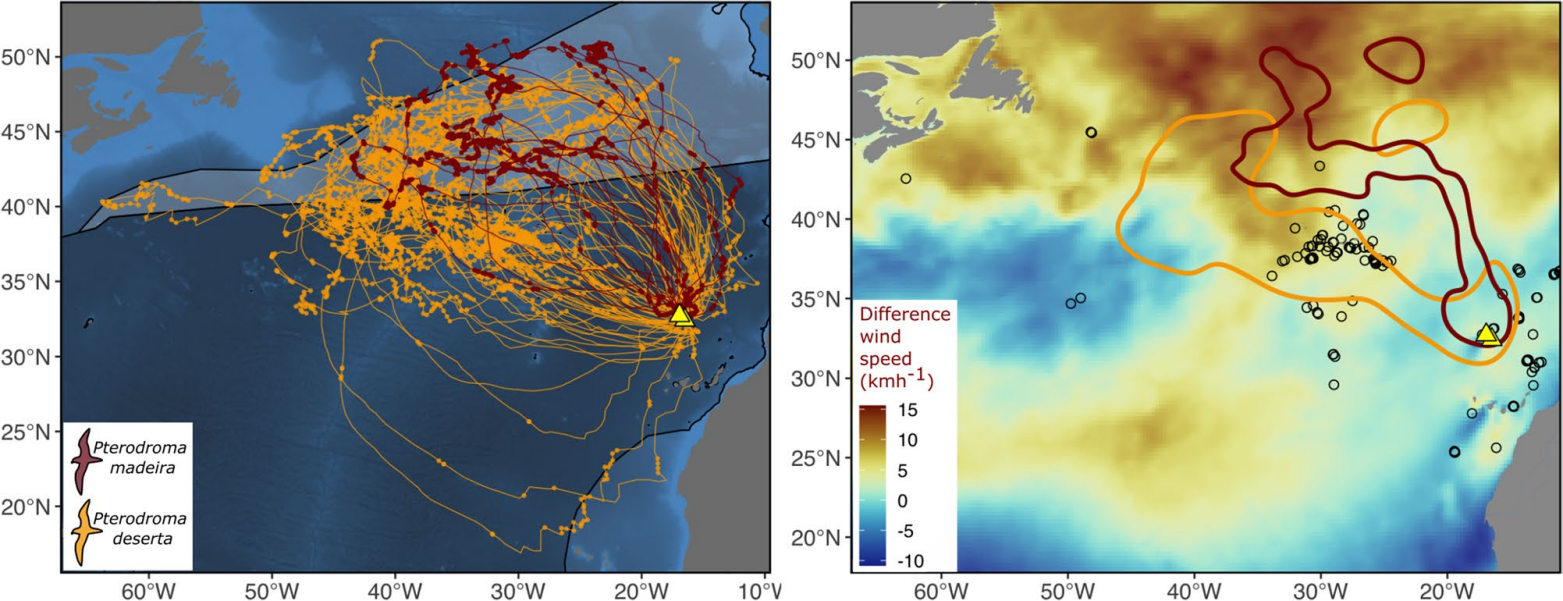
– Left panel: the foraging tracks of *Pterodroma madeira* (in red) and *P. deserta* (in gold) realised during the incubation phase of the breeding season (June-July and August to the first week of October, respectively). The dots represent the track locations classified as “search” by the Hidden Markov Models. The two breeding colonies are depicted by the yellow triangles. The transparent polygons represent the North Atlantic Drift (light polygon) and Central North Atlantic (dark polygon) mesopelagic ecoregions [50]. Right panel: the two species core breeding distribution, defined as the 50% Utilization Distribution contour computed based on GPS data. The circles show the location of seamounts. The map in the background shows the difference in wind speed (kmh^− 1^) between the incubation phase of *P. deserta* and *P. madeira*, considering the 2019 breeding season. We considered the incubation phases of each species encompassed in the 2019 GPS tracking database (28th of August– 6th of October for *P. deserta*; and 19th of June– 12th of July for *P. madeira*). Areas of increased wind speed are depicted in red, and represent locations where the wind is stronger during the incubation of *P. deserta* than during that of *P. madeira*. Areas of decreased wind speed are shown in blue

The Zino’s and Desertas petrel core distribution during incubation identified based on the GPS tracks was consistent with that estimated using the GLS data, and covered an area of approximately 880,000 km^2^ and 1.87 million km^2^, respectively (Fig. 3). The area most intensely used by breeding Zino’s petrels was located in the waters off the North-Northeast of the Azores archipelago, whereas the core breeding distribution of Desertas petrels encompassed a larger area towards the West-Northwest of the Azores.

### Isotopic niche

**Fig. 4:**
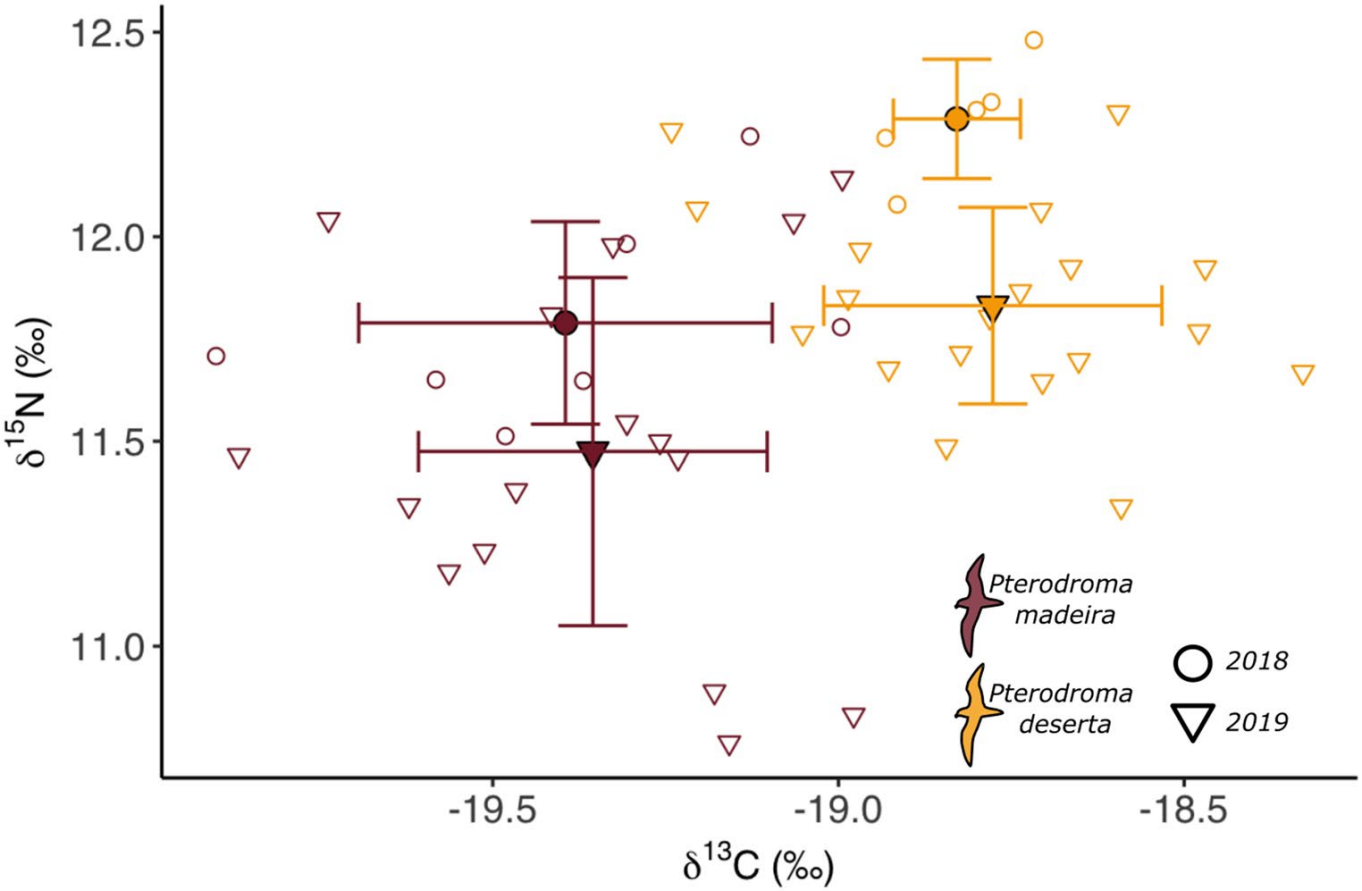
– Whole blood stable isotope values (δ^15^N and δ^13^C means +- SD, represented by the horizontal and vertical error bars) from *Pterodroma madeira* (in red, *n* = 24) and *P. deserta* (in gold, *n* = 25) during the respective incubation phases of the breeding seasons of 2018 (circles) and 2019 (triangles)

Overall, during incubation the Desertas petrel was significantly more enriched in ^15^N than the Zino’s petrel (F_1,47_=15.8, *P* < 0.001), and significant differences in ^15^N were found between the sampling years (F_1,47_=12.9, *P* < 0.001), with the 2018 season being significantly more enriched in ^15^N than the 2019 season for both species. Furthermore, the Desertas petrel was also significantly more enriched in ^13^C than the Zino’s petrel in both sampling years (F_1,47_=68.0, *P* < 0.001); no significant differences in ^13^C were detected between years (F_1,47_=0.31, *P* = 0.583) (Fig. 4).

### Behavioural classification

Desertas petrels spent more time in the transit state compared to Zino’s petrels, and undertook foraging trips that lasted for longer, covered a larger cumulative distance and reached areas further away from the colony (Table 1). As expected based on morphology [51], the larger Desertas petrel was slightly faster than the Zino’s petrel, in all states considered. Overall, considering all movement tracks, Desertas petrels experienced significantly stronger winds compared to Zino’s petrels (t-test, t = 4.52, d.f. = 21, *P* < 0.001). The average TWC experienced by the birds was also different, with Desertas petrels flying with a significantly stronger support from tail winds (t = 2.73, d.f. = 35, P = 0.01).

**Table 1:** – Characteristics (mean and sd, shown in brackets) of the central place (long) foraging tracks undertaken by *Pterodroma madeira* and *P. deserta* during the incubation phase of their breeding season, considering the entire track and the search and transit sections separately. The sampling units are the foraging trips and the sample size (“n”, shown in brackets) reflects the number of trips considered. The duration, cumulative distance and maximum distance were calculated considering all tracks (both collected at 1 h and at 2 h temporal resolution); all the other parameters were calculated using the tracks collected at the same temporal resolution (1 h)

Parameter	*Pterodroma madeira*		*Pterodroma deserta*	
	**All tracks (*n*** **= 12)**		**All tracks (*n*** **= 41)**	
Duration (days)	11.19 (3.19)		13.52 (3.58)	
Cumulative distance (km)	5656 (1359)		7864 (2212)	
Max distance from colony (km)	2043 (352)		2460 (579)	
	**Tracks 1 h resolution (*n*** **= 12)**		**Tracks 1 h resolution (*n*** **= 21)**	
Ground speed (kmh^-1^)	21.38 (2.82)		24.61 (3.08)	
Time in search state (%)	46 (9)		42 (12)	
Wind speed (kmh^-1^)	20.68 (2.62)		24.60 (3.25)	
Tail wind component (kmh^-1^)	4.65 (2.48)		6.55 (3.00)	
	**Search**	**Transit**	**Search**	**Transit**
Ground speed (kmh^-1^)	10.25 (1.34)	30.61 (2.86)	14.24 (2.03)	32.07 (1.63)
Wind speed (kmh^-1^)	20.49 (2.29)	20.95 (3.86)	25.85 (4.18)	23.73 (3.33)
Tail wind component (kmh^-1^)	4.47 (3.28)	4.70 (3.07)	7.83 (4.40)	5.64 (3.37)
Wind Δangle (°)	76.48 (8.38)	77.93 (7.54)	69.99 (11.99)	74.69 (10.31)
Sea surface temperature (°C)	18.20 (1.50)	19.44 (0.85)	22.50 (1.55)	22.50 (1.04)
Bathymetry (m)	-3651 (436)	-3875 (284)	-3841 (549)	-3977 (248)

### Habitat model

**Fig. 5:**
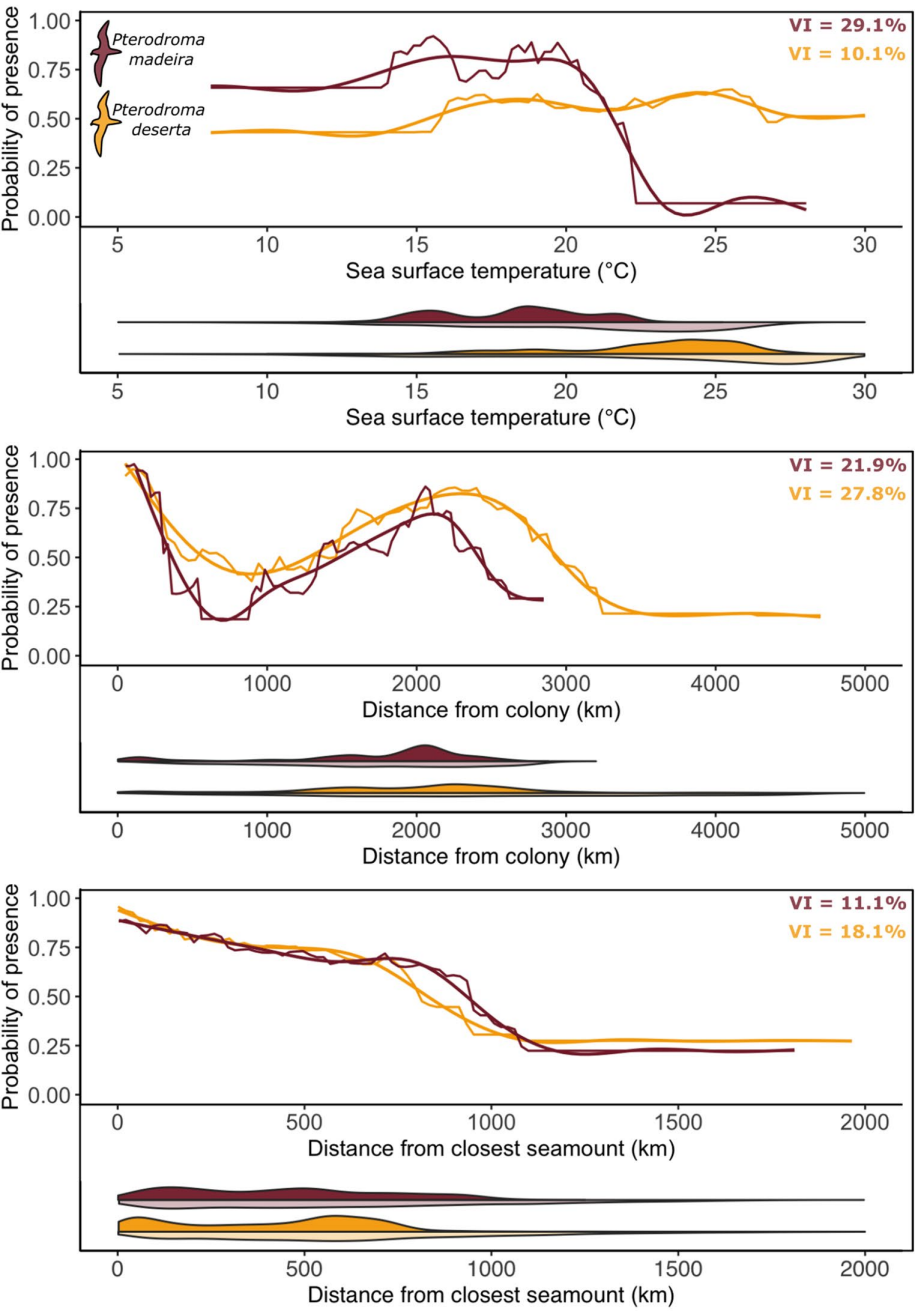
– Each panel shows: on top, the partial dependence plots, depicting the effect of a variable on the response, after accounting for the average effects of the other covariates in the model. We show the marginal effects (including a smooth representation of such effects, to aid interpretation) of the three most influential variables in the *habitat models* on the probability of presence of *Pterodroma madeira* and *P. deserta*, with respective relative variable importance (VI) in the top right corner. On the bottom, a violin plot depicting the density curves of the used (opaque, upper half of the violin) and available (transparent, lower half of the violin) explanatory variables. Results for *Pterodroma madeira* and *P. deserta* are shown in red and in gold, respectively

Overall, all the covariates included in the models had non-zero relative importance (supplementary information). Nevertheless, both species *habitat models* showed that three covariates played a dominant role in shaping the petrels’ probability of presence: SST (variable importance = 29.1% and 10.1% for Zino’s and Desertas petrels, respectively); distance from colony (variable importance = 21.9% and 27.8% for Zino’s and Desertas petrels, respectively); and distance from the closest seamount (variable importance = 11.1% and 18.1% for Zino’s and Desertas petrels, respectively) (Fig. 5). The probability of presence peaked for SST ∼16 °C and ∼25 °C for Zino’s and Desertas petrels, respectively. Additionally, the probability of presence of Zino’s and Desertas petrels peaked at around 2000 and 2400 km from the colony, respectively, and was higher in proximity of seamounts (Fig. 5). The *habitat models* for both species had good performance metrics (supplementary information), indicating that the distribution of foraging petrels can be adequately captured using the environmental variables considered.

### Wind model and track simulation

The *wind models* showed that the ground speed of both species is non-linearly affected by Δangle, wind intensity and their interaction. For both species, the transit ground speed peaked with quartering tailwinds (Fig. 1). These results, obtained with hourly data, were robust to the sampling rate of the datasets, as the predicted effect of wind on ground speed would be largely the same had we used data at 2 h resolution (supplementary information). The results of the simulation highlighted that both Zino’s and Desertas petrels could carry out their own foraging movements during the other species breeding season without significant changes to their trip duration (Fig. 1, “other season, own track”). However, had they performed the tracks of the other species, the duration of their trips would be significantly different (Fig. 1, “own season, other track”). Specifically, Desertas petrels would complete the simulated tracks in a significantly shorter time (Welch Two Sample t-test, t = 3.81, d.f. = 21, *P* = 0.001) whereas Zino’s petrels would take a significantly longer time (Welch Two Sample t-test, t = -5.79, d.f. = 12, *P* < 0.0001). Such changes in foraging movement duration are primarily driven by the intrinsic differences in the spatial extent of the two species trips, rather than by changes in ground speed attained along the simulated tracks. In other words, Zino’s petrels would take more time to complete the trips by Desertas petrels because they would have to cover more ground; conversely, Desertas petrels would take less time to complete the comparably shorter Zino’s petrel trips.

## Discussion

### Allochrony and spatial segregation

Both Zino’s and Desertas petrels are widely distributed across the Atlantic Ocean throughout the yearly cycle. Between May and October, they used a vast pelagic region, part of the North Atlantic Current and mid-Atlantic Subpolar frontal system, a major seabird hotspot [52] characterized by enhanced productivity at a large scale. The areas most intensely used by the breeding petrels were within two large mesopelagic ecoregions (see below and Fig. 3). During the winter, non-breeding petrels widely dispersed across a vast marine region comprising the North and South Equatorial Current and the southward extension of the Brazilian Current (only used by the Desertas petrel). Additionally, one individual Zino’s petrel used oceanic waters in the Central South Atlantic.

The spatial distribution of Zino’s and Desertas petrels overlaps substantially, both during the breeding and non-breeding seasons. If the petrels were to breed at the same time and maintain their space use patterns, their realised spatial overlap would effectively increase. Therefore, the observed 2-month asynchrony in their breeding phenology contributes to the emergence of segregation in the petrels’ year-round spatial distribution. Clear patterns of ecological segregation and potential drivers of allochrony emerge when focussing on the movement and stable isotopes datasets collected during the breeding season– the most energetically demanding phase of their life cycle, when petrels are constrained to live in close geographical proximity.

### Allochrony and foraging niche segregation

During incubation, Zino’s and Desertas petrels exhibit significant isotopic niche partitioning. Differences in blood stable isotope values are indicative of the two species feeding in different ocean habitats, characterised by different biogeochemical properties and, likely, different prey compositions. In this context, in line with the findings of other studies [15], the substantially longer and more robust bill structure may enable Desertas petrels to target larger prey and, potentially, feed at a higher trophic level than Zino’s petrels.

The core spatial usage of the petrels largely fell within two distinct mesopelagic ecoregions [50] (Fig. 3) with different physical-chemical conditions and distinct mesopelagic faunal compositions, corroborating the findings above on isotopic niche divergences. Zino’s petrels almost exclusively used the North Atlantic Drift, a transition ecotone with boreal and subtropical species expanding eastwards following the Gulf Stream, whereas Desertas petrels primarily used the Central North Atlantic, a vast region of warmer and more stable temperature-salinity-oxygen conditions. The usage of these areas may, in turn, underpin the different SST preferences exhibited by the species and, ultimately, dietary partitioning. The different habitat preferences highlighted by the *habitat models* provide further evidence that the petrels may target different prey heterogeneously distributed in pelagic habitats. The key variables driving the foraging activity of Zino’s and Desertas petrels were SST (with different preference peaks for the different species and a higher relative importance for Zino’s rather than Desertas petrels), distance from the colony and distance from seamounts. Physical-chemical properties of oceanic waters (such as temperature and oxygen) and bathymetric features such as seamounts (which may bring nutrient rich waters towards the surface or modulate the vertical migration of mesopelagic communities [53]) affect the foraging activity of several pelagic seabirds, including other gadfly petrels [34, 54].

Little is known about the diet of Zino’s and Desertas petrels but, similarly to other gadfly petrels [31, 55], they are generalist predators feeding on mesopelagic prey species (own unpublished data). Similarities in the foraging ecology, flight behaviour and moulting schedule of the petrels are indicated by consistent patterns (with a ∼ 2-month lag) in their yearly at-sea activity. During the energetically demanding breeding season, they spend more time in flight, whereas during non-breeding both species spend more time sitting on the water, presumably due to lower energetic requirements and to the sequential moult of their primary feathers (Fig. 1) [56]. Freed from their breeding constraints, they modulate their flight activity in relation to the moon cycle, spending more time in active flight during nights closer to full moon, in line with the findings for other seabirds [57].

It is difficult to determine whether the patterns of foraging niche segregation highlighted by stable isotopes and *habitat models* are driven by foraging specialisation or by changes in the underlying resource availability between the breeding seasons of the two species. Nevertheless, based on our results, the petrel breeding allochrony seems to be sustained by adaptations to local environmental conditions and different foraging resources rather than by adaptations to the windscape (see below). On the one hand, breeding Zino’s petrels may exploit the large algal bloom and enhanced productivity characteristic of the North Atlantic Drift in late spring. On the other hand, phylogenetic analyses revealed that Desertas petrels and Cape Verde petrels (*Pterodroma feae*) are more closely related to each other than they are to Zino’s petrels [30], suggesting that Desertas petrels arrived on the Madeira archipelago following a colonization event by an ancestral population from Cape Verde. The preference for warmer waters by Desertas petrels is therefore somewhat suggestive of Desertas petrels modulating their breeding schedule to exploit a SST niche consistent with that of the ancestral population. Ultimately, our findings are indicative of a spatial and temporal diversity of the (very poorly understood) mesopelagic communities [58], which are a dominant component of the oceanic food web and can sustain foraging niche divergences even in generalist pelagic seabirds.

### Allochrony and windscape

Despite the different morphologies and, importantly, despite the marked differences in the windscape available during the two breeding seasons (Fig. 3), the *wind models* and simulations do not support the hypothesis that gadfly petrels modulate their breeding phenology to realise central-place foraging movements when wind conditions are most favourable. As for other dynamic soarers [24], crosswind to down-wind flight seems to be the preferred flight mode for Zino’s and Desertas petrels and the one that maximises the two species’ ground speed. Regarding the ground speed values presented in this work, it is important to highlight that they are likely to be an underestimate of the real speed attained by the petrels, as our calculations do not account for the changes in direction and for the sinuosity of the dynamic soaring flight within the hourly movement steps. Due to a similar functional relationship between ground speed and wind, the flight performance attained by the petrels would not change significantly had they experienced the windscape throughout the other species’ tracks or during the other species’ breeding season. Nevertheless, the petrels exploited different wind conditions and performed trips of different spatio-temporal extent. As we discuss below, it is possible that intrinsic species-specific differences in morphology may affect breeding allochrony in ways not captured by our simple *wind models* that only focus on ground speed.

### Intrinsic drivers of foraging ecology

The foraging movements of the petrels were intrinsically different in terms of their temporal duration and distance covered, with the larger Desertas petrels carrying out longer tracks, covering more distance in a longer period of time. Body mass influences energy management of seabirds, particularly in relation to the prolonged period of fasting during incubation stints before being relieved by the partner [59]. As both the metabolic costs and the capability of storing energy reserves increase with body size, but the latter increases faster, fasting endurance rapidly increases with body mass in seabirds [38]. Moreover, the air temperature and elevation at the nesting ground may also play a role: Zino’s petrels breeding in spring in the central mountain massif of Madeira (∼ 1800 m above sea level) are subject to lower temperatures than those experienced by Desertas petrels on Bugio Island (∼ 300 m above sea level). This may result in a higher resting metabolic rate for Zino’s petrels than Desertas petrels, further reducing their fasting endurance. Thus, compared to the smaller and lighter Zino’s petrels, Desertas petrels may be better able to sustain longer incubation shifts, releasing partners to forage over equally longer periods, allowing them to carry out some of the longest foraging movements recorded in any breeding animal [25] and explore areas that may be inaccessible to Zino’s petrels during the breeding season.

In *Procellariiformes* and other seabird taxa, differences in functional traits such as body mass and flight morphology set physiological constraints to the operational and tolerable wind speeds [18]. Throughout their breeding seasons, the petrels in this study used significantly different wind conditions and were exposed to largely different wind niches. Thus, despite a largely similar (simulated) ground speed attained in both breeding seasons, on the one hand the higher wing loading may enable the heavier Desertas petrels to exploit the stronger North Atlantic wind conditions occurring later in the year (Fig. 3) and sustain longer foraging movements. Being positively correlated with energy expenditure, the higher wing loading may also imply that Desertas petrels need stronger winds to sustain soaring and buffer this increased energetic cost throughout their central-place-foraging tracks. On the other hand, the lighter Zino’s petrels may time their reproductive schedule to complete breeding before the onset of the stronger winds, avoiding the resulting higher aerodynamic force on their wings and increased wind drift [60] that may cause them to fail to return to their nest on time to relieve their partner.

## Conclusions

The allochrony in the breeding cycles of the two species is underpinned by patterns of foraging niche partitioning during the breeding season. Furthermore, the petrels performed intrinsically different foraging movements exploiting largely different wind niches. As expected based on biomechanics, the heavier Desertas petrels used stronger winds and performed longer foraging movements, whereas Zino’s petrels realised shorter foraging movements under the weaker wind conditions earlier in the year.Foraging niche segregation reducing historical competition (rather than ongoing, given the present low population sizes) may have allowed these similar species to coexist in sympatry. Ultimately, our work suggests that an interplay between morphology, fasting endurance and foraging trip regulation may be an important (and yet overlooked) mechanism shaping the foraging ecology and promoting patterns of ecological segregation in sympatric species.

### Electronic supplementary material

Below is the link to the electronic supplementary material.

Supplementary Material 1

## Data Availability

The GPS and GLS datasets are available in the BirdLife Seabird Tracking Database. The data and R scripts to reproduce the analysis are available from the Figshare digital repository (https://figshare.com/authors/Francesco_Ventura/7066628).

## Abbreviations

BRT: Boosted Regression Tree
GAMM: Generalised Additive Mixed Model
GLS: geolocator-immersion loggers
HMM: discrete-time hidden-Markov-model
SST: sea surface temperature
TWC: tail wind component
UD: utilization distribution
VI: relative variable importance
Δangle: wind direction relative to bird movement direction
