# Supplementary material for: Allochrony is shaped by foraging niche segregation rather than adaptation to the windscape in long-ranging seabirds
Source: Mov Ecol. 2024 Apr 2;12:27. doi: 10.1186/s40462-024-00463-z (PMC10988818; doi:10.1186/s40462-024-00463-z)
Supplement: Supplementary file 1 — Supplementary Material 1 [file 40462_2024_463_MOESM1_ESM.pdf]

Supplementary Information for

Allochrony is shaped by foraging niche segregation rather than adaptation to the windscape in a long-ranging seabird

Ventura, Francesco[1*]; Granadeiro, José Pedro[2]; Catry, Paulo[3]; Gjerdrum, Carina[4]; De Pascalis, Federico[5]; Viveiros, Filipe[6]; Silva, Isamberto[6]; Menezes, Dilia[6]; Paiva, Vítor H.[7]; Silva, Mónica C.[8]

[1]Biology Department, Woods Hole Oceanographic Institution, Woods Hole, MA, USA
[2]CESAM, Departamento de Biologia Animal, Faculdade de Ciências, Universidade de Lisboa, Campo Grande, 1749-016 Lisboa, Portugal
[3]MARE – Marine and Environmental Sciences Centre / ARNET – Aquatic Research Network, Ispa – Instituto Universitário, Rua Jardim do Tabaco 34, 1149-041 Lisboa, Portugal
[4]Canadian Wildlife Service, Environment and Climate Change Canada, Dartmouth, Nova Scotia B2Y 2N6, Canada
[5]Area Avifauna Migratrice, Istituto Superiore per la Protezione e la Ricerca Ambientale (ISPRA), Ozzano dell'Emilia, Italy
[6]Parque Natural da Madeira, Quinta do Bom Sucesso, Caminho do Meio, 9050-251, Funchal, Madeira, Portugal
[7]University of Coimbra, MARE-Marine and Environmental Sciences Centre / ARNET – Aquatic Research Network, Department of Life Sciences, Calçada Martim de Freitas, 3000-456, Coimbra, Portugal
[8]Centre for Ecology, Evolution and Environmental Changes (cE3c), Departamento de Biologia Animal, Faculdade de Ciências, Universidade de Lisboa, Campo Grande, 1749-016 Lisboa, Portugal

*Corresponding author: Francesco Ventura
Email: fraventura.92@gmail.com

GPS tracking data

GPS tracks were collected using Pathtrack GPS devices programmed to store one location every 1 or 2 hours. The loggers were either 3.0 g or 7.4 g and therefore represented (with added tape) less than 3% of the average body mass. They were attached with Tesa® tape on the four central tail feathers and retrieved from the tracked birds when they returned to the nest. Both species performed both long and short trips at-sea (fig. 1). The role of these short movement bouts during incubation is unclear and potentially not primarily linked to foraging. To quantitatively distinguish between long and short tracks, we used k-means clustering (with 2 clusters for "short" and "long" trips) and classified each movement track based on their maximum distance from the colony and total time at-sea using the Hartigan and Wong algorithm. The short movement bouts, limited both in spatial and temporal scale, represented only 13% (*Pterodroma madeira,* hereafter PMAD) and 11% (*P. deserta,* hereafter PDES) of the total recorded time spent at-sea by the tracked animals. We removed the short trips from the analysis and retained a total of: 16 long foraging trips for PMAD (7 trips in 2018; 9 in 2019); and 43 long tracks for PDES (1 in 2015; 8 in 2016; 18 in 2017 and 16 in 2019).

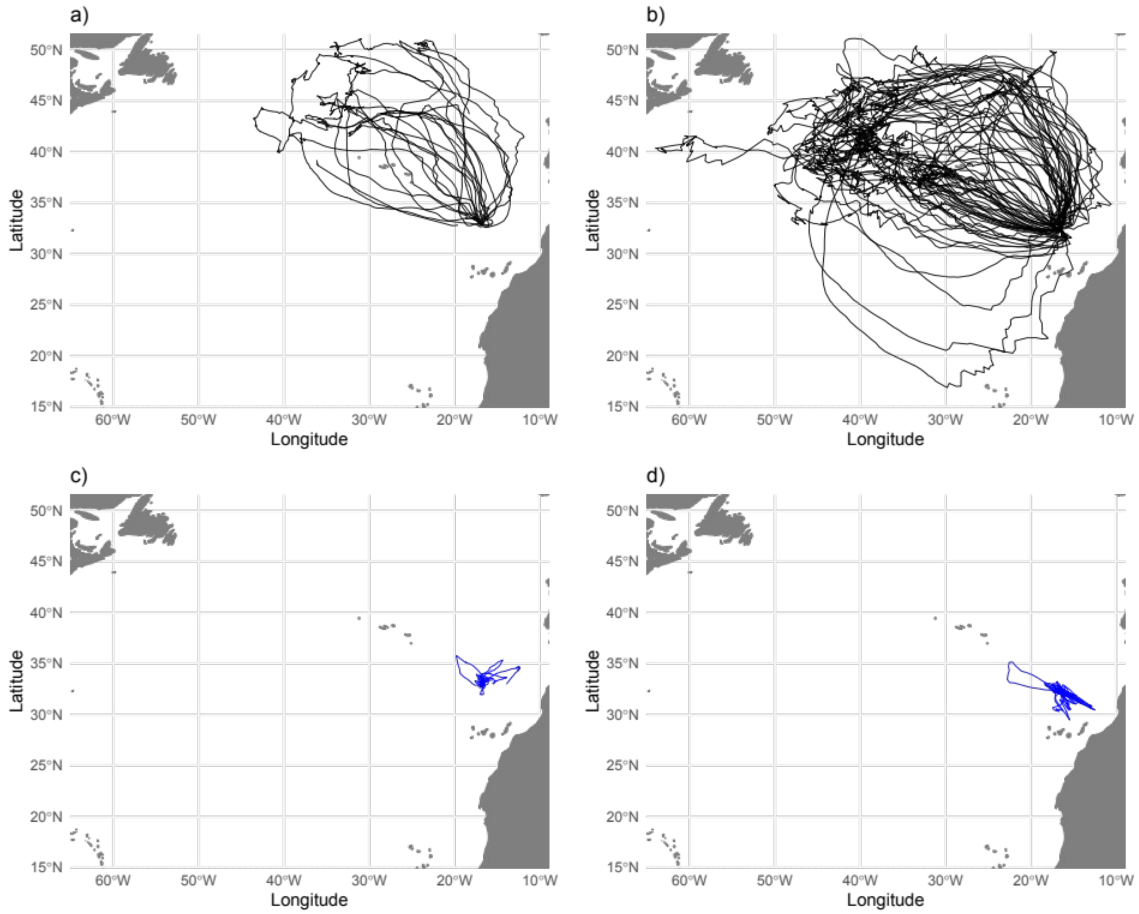

Fig. 1 – The central place foraging movement bouts collected for *Pterodroma madeira* (panel a, c) and *P. deserta* (panel b, d). Black tracks (panel a, b) represent those that were classified as "long" by the k-mean clustering analysis; blue tracks (panel c, d) are the "short" trips.

GLS data processing

Light intensity data recorded by the GLS were analysed and visually inspected using the TransEdit2 software (British Antarctic Survey) to determine the timing of twilight events, under a light threshold equal to 10. To compute the positions of the animals at sea, we used the

probabilistic algorithm embedded in the R package probGLS [1]. To improve the performance of the algorithm results, we used a land mask. Furthermore, we associated the dataset containing the timing of twilight events to that storing the wet/dry activity of the petrels, which enabled us to set a "dry" (i.e. in flight) and "wet" speed filters. We assumed optimal travel speed of 12 m/s (sd = 5 m/s) and max speed = 34 m/s when the petrels were in flight; and 1 m/s (sd = 1.3 m/s) and max speed = 4 m/s when the petrels were on the water. We assumed that the error around the timing of twilight events was longnormally distributed, with shape = 2.49 and scale = 0.94 and delay (the error distribution used as default in the probGLS package). The probabilistic algorithm simulated 100 movement tracks using 1000 number of particles for each location cloud, testing a range of sun elevation angles between -4 and -1. For 7 days before and after the spring and fall equinox (when the latitude estimates are particularly unreliable), the algorithm drew random latitudes from a boundary box between lon [-80,40] and lat [-50,50]. The most likely movement path was computed as the geographic median of the 100 simulated movement paths.

GLS data processing – activity data

To describe the year-round activity patterns of the petrels, we considered the wet/dry periods recorded by the GLS. The devices sampled the immersion status every 30 seconds, and recorded the number of wet samples every 10 minutes throughout the deployments. The daily proportion of saltwater immersion was calculated as the daily number of wet samples over the total daily number of samples (n= 2880, i.e., the total number of samples collected every 30 seconds in 24 hours).

GLS data processing – proportion of moon illumination

The proportion of moon illumination for every day throughout the GLS deployment was calculated using the "lunar" package in R [2]. We used the R function "lunar.illumination", adapted from the R4MFCL project (https://code.google.com/archive/p/r4mfcl/) developed by the Secretariat of the Pacific Community.

Stable Isotopes Analysis

Blood was extracted from the tarsal vein of incubating individuals upon first capture. Whole blood samples (of up to 150 μl per bird) were transported in a cooler in the field and stored at -20ºC. In the lab they were dried at 50 °C for approximately 48h and subsequently ground into powder. Sub-samples of homogenous blood of 1mg (±0.1) were weighed in tin capsules. Stable isotope analyses of whole blood were carried out at the Stable Isotopes and Instrumental Analysis Facility (SIIAF), Faculdade de Ciências, Universidade de Lisboa, using continuous flow isotope mass spectrometry on a Sercon Hydra 20-22 (Sercon, UK) spectrometer, coupled to a EuroEA (EuroVector, Italy) elemental analyser. The reference materials used were IAEA N1, IAEA N2 and USGS26, and Glucose BCR no. 657, IAEA-CH7 and IAEA-C3 [3]. The laboratory QC check used was Casein. Precision was calculated using values from three to nine replicates of laboratory standard materials (Casein ISO) interspersed among samples in every batch analysis. The d15N values of the different batches varied between 5.62 and 5.72, whereas the d13C values varied between -20.53 and -20.65. Internal laboratory standards assessment indicated that the measurement error was ≤0.1 ‰ for $\delta^{13}C$ and $\delta^{15}N$.

Behavioural classification

We used a state-space generalized multivariate hidden Markov modelling (HMM) framework to identify the behavioural states ("searching" or "transit") of each petrel along the track, based on the observed step lengths and turning angles. Step length (i.e., ground speed) is intrinsically dependent on the tail wind component (TWC) experienced by the birds and, particularly so when in the transit state, birds travelling with increased support of tail winds should be capable of attaining higher ground speeds. Hence, to increase the biological realism of the HMMs, we modelled the mean parameter of the state-dependent probability distribution of step length as a function of TWC using the DM (design-matrix) argument formulation embedded in the momentuHMM package in R [4]. In so doing, we simultaneously accounted for the TWC effect on the on the mean of the state- dependent step length distributions for both states (fig. 2-3).

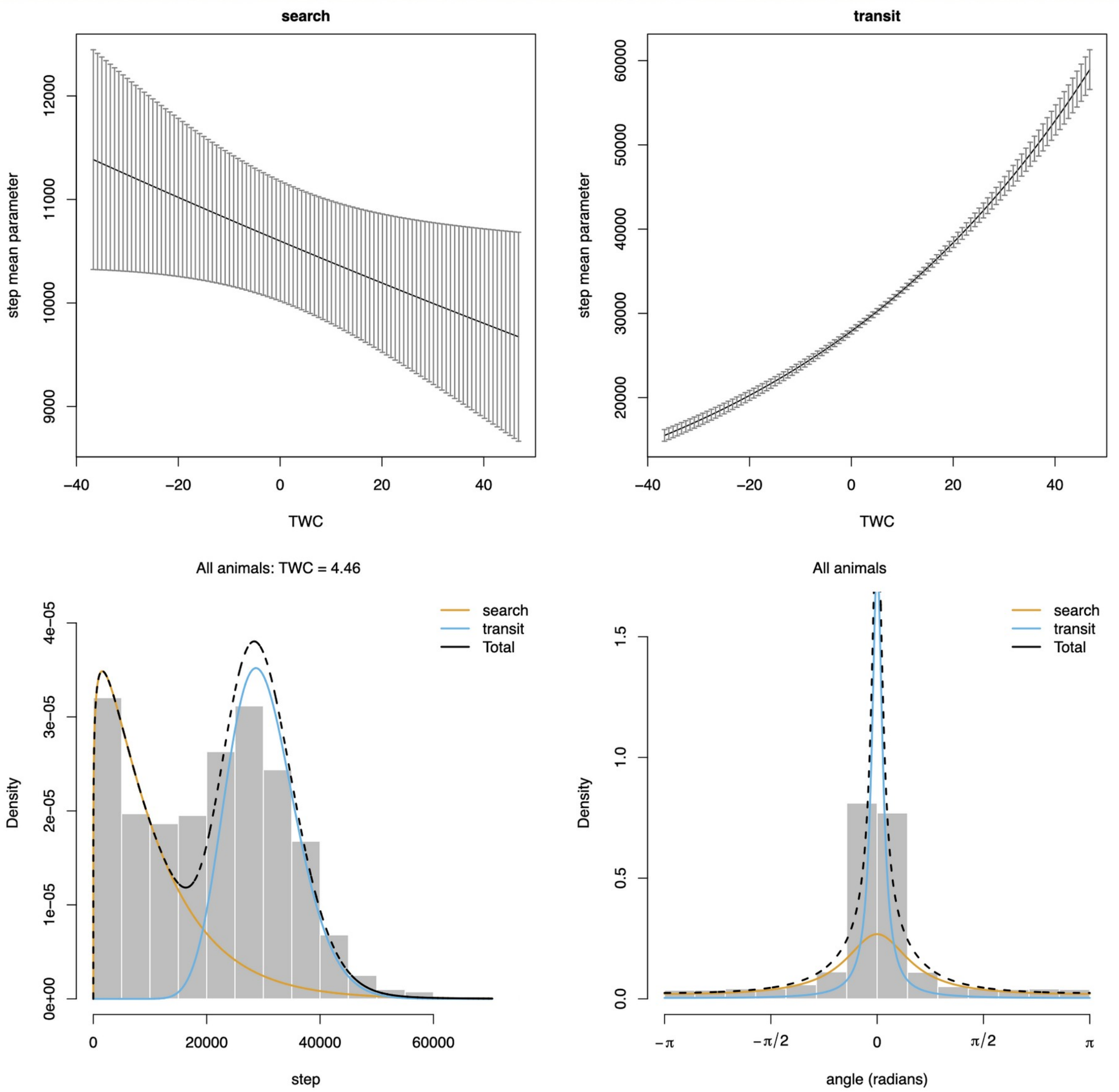

Fig. 2 – Results of the *Pterodroma madeira* HMM. The panels above show the estimated effect of TWC (with 95% CI) on the step length mean parameter for the "search" ("left) and "transit" (right) states. The bottom panels represent histograms of the step length and turning angle data, with the solid lines showing the estimated state-dependent probability distributions. As TWC in the model design-matrix affects both states' step length mean parameter, the step length probability distributions are calculated for mean TWC values.

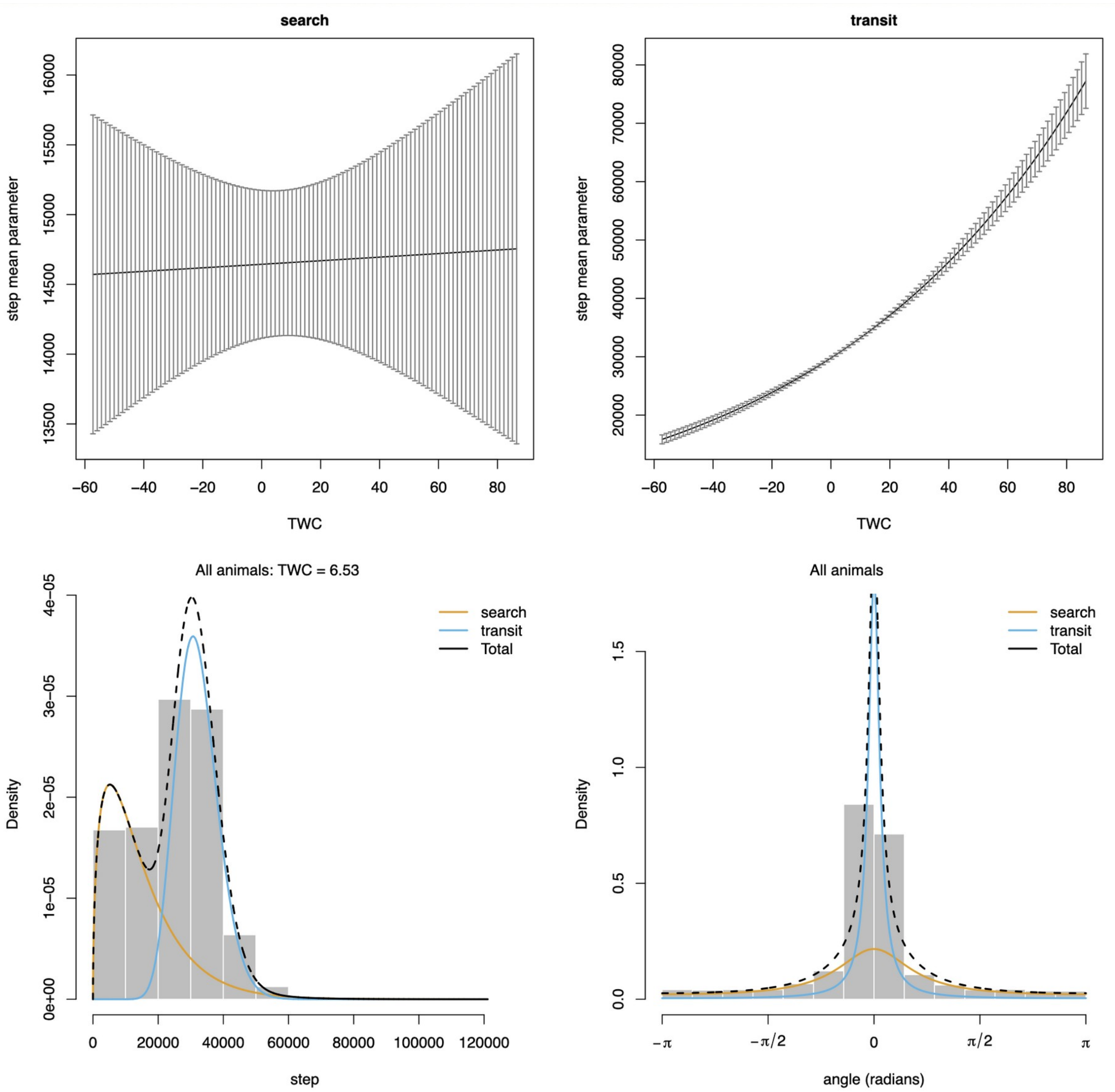

Fig. 3 – Results of the *Pterodroma deserta* HMM. The panels above show the estimated effect of TWC (with 95% CI) on the step length mean parameter for the "search" ("left) and "transit" (right) states. The bottom panels represent histograms of the step length and turning angle data, with the solid lines showing the estimated state-dependent probability distributions. As TWC in the model design-matrix affects both states' step length mean parameter, the step length probability distributions are calculated for mean TWC values.

Environmental covariates for boosted regression trees

The following environmental variables were included in the boosted regression trees (BRTs) as we hypothesized them to affect the petrel probability of presence.

Wind speed. Positive effect of wind on the movement and search efficiency of the petrels. Wind also enhances water mixing, resulting in localised areas of higher productivity. We expect higher probability of presence in windier locations.

Depth. Different prey distributed in shelf and oceanic waters. Gadfly petrels mainly consume mesopelagic species. The animals are predicted to search in the bathymetric domain with higher preferred prey availability.

Slope. Slope areas are regions of water mixing, where nutrient-rich waters are forced towards the surface. We expect the animals to concentrate in the more productive waters over the shelf (if preferred prey predictably aggregate over the slope areas).
Sea surface temperature. Different sea surface temperatures and different physical-chemical properties of oceanic waters may structure mesopelagic communities and shape mesopelagic faunal composition, affecting the probability of presence of the petrels.
Sea surface temperature gradient. Higher gradients are indicative of frontal locations, which are known to be associated with enhanced productivity and prey aggregation.
Sea surface height above sea level. Variable capturing convergence/divergence of water masses. Low sea surface heights indicate divergence zones and upwelling of nutrients towards the surface.
Eddy kinetic energy. Eddies modulate structure of pelagic ecosystems. Waters characterised by higher eddy activity are associated with enhanced primary production and prey aggregation. We predict the petrel probability of presence to be higher in locations with higher eddy kinetic energies.
Chlorophyll A concentration. Index of primary productivity. More productive waters support higher densities of potential prey. We expect higher probability of presence in more productive waters with higher prey availability.
Distance from colony. Determines habitat availability for central place foragers.
Distance from seamounts. Near seamounts, physical forcing of nutrient rich waters towards the surface. The areas closer to seamounts grant the animals more foraging opportunities. We predict an increased probability of presence in proximity of seamounts.
Density ocean mixed layer thickness. The depth of the mixed layer describes the physical structure and the stratification of the water column. We expect higher probability of presence in waters characterised by deeper mixed layers, as they are characterised by enhanced water mixing and higher availability of nutrients.
Mass content of "epipelagic", "migrant upper mesopelagic " and "highly migrant lower mesopelagic" micronekton. SEAPODYM (http://www.seapodym.eu) model output of epipelagic and mesopelagic biomass. We predict the petrels to concentrate their search in areas with higher predicted micronekton biomass.

Variable	Spatial resolution	Temporal resolution	Source
Wind speed (km/h)	0.25°	6 hours	ECMWF ERA-5 database (https://cds.climate.copernicus.eu/cdsapp). Calculated as: Wind speed = $(U_{wind}^2 + V_{wind}^2)^{0.5}$
Bathymetric depth (m)	0.004°	NA	GEBCO bathymetry map (https://www.gebco.net)
Bathymetric slope (°)	0.004°	NA	Calculated from depth raster using the 'terrain' function from raster [7] package
Sea surface temperature (°C)	0.01°	daily	NOAA (https://coastwatch.pfeg.noaa.gov/erddap/griddap/jplMURSST41)
Sea surface temperature gradient (°)	0.01°	daily	Calculated from SST rasters using the 'terrain' function from raster [7] R package
Sea level anomaly (m)	0.25°	daily	E.U. Copernicus Marine Service Information (https://resources.marine.copernicus.eu/product-detail/SEALEVEL_GLO_PHY_L4_MY_008_047/)
Eddy kinetic energy (m^2/s^2)	0.25°	daily	E.U. Copernicus Marine Service Information (https://resources.marine.copernicus.eu/product-detail/SEALEVEL_GLO_PHY_L4_MY_008_047/). Calculated from geostrophic current velocity as: EKE = $0.5*(U_a^2 + V_a^2)$

Chlorophyll A concentration (mg/m^3)	0.04°	daily	E.U. Copernicus Marine Service Information (https://data.marine.copernicus.eu/product/OCEANCOLOUR_GLO_BGC_L4_MY_009_104/download?dataset=cmems_obs-oc_glo_bgc-plankton_my_l4-gapfree-multi-4km_P1D)
Distance from colony (km)	NA	NA	Calculated as the great circle distance of each point from the colony, using the function spDistsN1 from the sp package [5] in R.
Distance from seamounts (km)	0.05°	NA	"Nearest neighbour" distances of each pixel from the closest seamount computed for a 0.05° x 0.05° resolution raster. Locations of seamounts were extracted from the Wessel's global seamounts map [6], considering seamounts that are at a depth between 0 and 500 m)
Density ocean mixed layer thickness (m)	0.08°	daily	E.U. Copernicus Marine Service Information (https://data.marine.copernicus.eu/product/GLOBAL_ANALYSISFORECAST_PHY_001_024/description)
Mass content of epipelagic micronekton (wet weight in sea water, g/m^2)	0.08°	daily	SEAPODYM dynamical population model output (http://www.seapodym.eu). Available at E.U. Copernicus Marine Service Information (https://data.marine.copernicus.eu/product/GLOBAL_MULTIYEAR_BGC_001_033/description)
Mass content of migrant upper mesopelagic micronekton (wet weight in sea water, g/m^2)	0.08°	daily	SEAPODYM dynamical population model output (http://www.seapodym.eu). Available at E.U. Copernicus Marine Service Information (https://data.marine.copernicus.eu/product/GLOBAL_MULTIYEAR_BGC_001_033/description)
Mass content of highly migrant lower mesopelagic micronekton (wet weight in sea water, g/m^2)	0.08°	daily	SEAPODYM dynamical population model output (http://www.seapodym.eu). Available at E.U. Copernicus Marine Service Information (https://data.marine.copernicus.eu/product/GLOBAL_MULTIYEAR_BGC_001_033/description)

Boosted regression trees – relative variable importance

Pterodroma madeira		***Pterodroma deserta***	
**Variable**	**Relative importance (%)**	**Variable**	**Relative importance (%)**
Sea surface temperature	29.13	Distance from colony	27.75
Distance from colony	21.88	Distance from seamounts	18.12
Distance from seamounts	11.09	Sea surface temperature	10.55
Sea level anomaly	6.69	Chlorophyll A concentration	7.04
Bathymetric depth	5.99	Eddy kinetic energy	5.89
Chlorophyll A concentration	4.57	Sea level anomaly	5.83
Wind speed	4.34	Bathymetric depth	3.98
Eddy kinetic energy	4.08	Wind speed	3.88
Density ocean mixed layer	2.81	Density ocean mixed layer	3.68

thickness		thickness	
Sea surface temperature gradient	2.78	Epipelagic micronekton	3.33
Highly migrant lower mesopelagic micronekton	2.04	Highly migrant lower mesopelagic micronekton	3.25
Epipelagic micronekton	1.86	Sea surface temperature gradient (°)	2.77
Bathymetric slope	1.52	Migrant upper mesopelagic micronekton	2.12
Migrant upper mesopelagic micronekton	1.24	Bathymetric slope	1.80

Boosted regression trees – parametrization and evaluation of model performance

BRTs were fit to the data with the bernoulli loss function, using the gbm.step function from the dismo package [8] in R, adopting the following hyper-parametrization: tree complexity = 3; learning rate = 0.05; bag fraction = 0.6. The optimal number of boosting trees was assessed using k-fold cross validation, and estimated at 2300 and 2100 trees for PMAD and PDES, respectively. The predictive performance of the BRTs was evaluated using K-fold cross-validation metrics [9]. To do so, we randomly assigned the dataset points to k (10) groups; trained the BRT on k-1 (9) groups and tested it on the group left out. Then, comparing the observed data with the predicted values, we calculated the % of deviance explained by the model; the area under the receiver operating curve ("AUC", with values closer to 1 indicating better fit); and true skill statistics ("TSS"). The latter was calculated as TSS = sensitivity + specificity -1, ranging between -1 to 1, with 1 indicating perfect agreement between observations and predictions, whereas values of zero or less indicates a performance no better than random. The habitat models for both species had good performance metrics, with high deviance explained (mean deviance explained = 87% and 64% for PMAD and PDES, respectively), AUC (mean AUC = 0.98 and 0.93 for PMAD and PDES, respectively) and TSS (on average = 0.86 and 0.72 for PMAD and PDES, respectively), indicating that the distribution of foraging petrels can be adequately captured using the environmental variables considered.

Wind models sensitivity analysis

We investigated the effect of sampling rate on the predicted relationship between ground speed and wind. To do so, we subsampled the hourly data fed to the wind models (see the main text) at 2h resolution; calculated bearing and step length; and associated wind speed and wind direction relative to the bird direction. The results obtained with the subsampled dataset (shown below in fig.4) are consistent with those presented in the main text, although as expected the observed mean travelling speed of the subsampled data (PMAD: 30.0 km/h ± 8.0 km/h; PDES: 31.6 km/h ± 7.3 km/h) was slightly lower than that measured for the hourly data (PMAD: 30.3 km/h ± 8.3 km/h; PDES: 32.0 km/h ± 7.5 km/h).

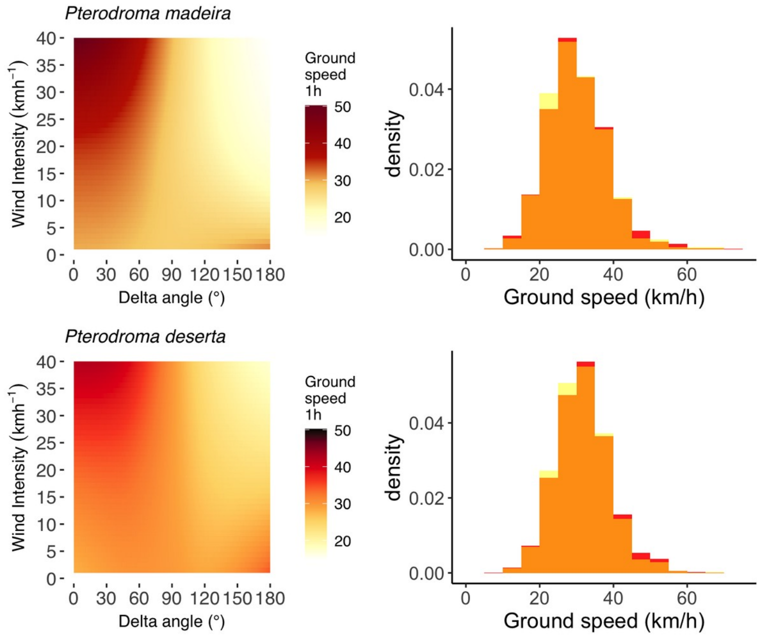

Fig. 4 – Left column: heatmap showing the relationship between hourly ground speed, wind intensity and direction relative to bird heading ("delta angle"), predicted by the wind models. Here, the wind models were fit to data subsampled at 2h resolution. The results are consistent with those presented in the main text. Right column: histograms showing the observed ground speed in the hourly data (in red, used in the main text) vs. the subsampled 2h resolution data (in yellow). As expected, the observed mean travelling speed of the subsampled data (PMAD: 30.0 km/h ± 8.0 km/h; PDES: 31.6 km/h ± 7.3 km/h) was slightly lower than that measured for the hourly data (PMAD: 30.3 km/h ± 8.3 km/h; PDES: 32.0 km/h ± 7.5 km/h).

Track simulation

We adopted a simulation framework to investigate whether each species achieved highest flight performance (in terms of temporal duration of the tracks and ground speed) by exploiting the windscape of their own breeding season, or whether they may have sustained equivalent foraging flights during the other species breeding season. We limited our analysis to the season 2019, when breeding tracks of both PMAD and PDES were available. We followed the methodology in [10] and considered only the transit bouts of the tracks. Thus, the last point of each transit section interrupted by search locations was artificially connected to the first point of the subsequent transit bout by a beeline segment. These connecting segments were only computed in order to preserve the trip configuration, but they were discarded prior to calculating the final trip metrics. The values yielded by the simulation framework therefore only refer to the 'transit' section of the tracks, rather than to the overall routes.

In the simulation, we did not consider the whole breeding season of the two species. Rather, as our data comprised incubation tracks, we only focussed on the incubation phase of each species breeding season. Moreover, we only considered the dates comprised in our tracking dataset. Thus, the PMAD season considered for the simulation was defined between the 15th of June to 7th of July; the PDES season was defined between the 15th of August to 7th of October. As explained in the main text, based on the species-specific relationship between ground speed and wind estimated by the respective *wind model*, we calculated the ground speed attained by each species considering the scenarios outlined below.

(a) Focal species performing the tracks of the other species during its own breeding season. Here, for example, we let PMAD carry out foraging tracks during the incubation phase of their own breeding season, but forcing them to realise the tracks of PDES. In other words, we

simulated that PMAD carried out each of the observed movement tracks of PDES; and each movement track was initialised every day between the 15th of June to 7th of July.

(b) Focal species undertaking its observed tracks during the breeding season of the other species. Here, for instance, we let PMAD carry out their own tracks during the PDES season. To do so, we artificially changed the departure date of each tracks using the time stamp of the PDES season. In other words, we simulated a new PMAD track for each day between the 15th of August and the 7th of October.

For all simulation scenarios described above, the duration and ground speed attained throughout each simulated trip was calculated as follows. Given the initial timestamp and the start location "s" of each trip, we calculated the time needed for the bird to travel to the next point "s + 1" based on the ground speed predicted by the wind model for the given the wind conditions (Δangle and wind intensity) experienced at "s". For each species, this calculation was based on the respective species-specific *wind model* (see main text). In this step, when calculating the predicted ground speed, we assumed that the maximum ground speed achievable by the focal species was equal to the maximum observed ground speed in the respective tracking dataset. The time of arrival at "s + 1" and the local wind conditions at that time were then calculated. This protocol was repeated for all locations along each simulated trip. For each scenario, we computed the overall duration and ground speed averaged across all simulated tracks and compared them to the average duration and speed of the real tracks (i.e., the observed tracks carried out by the focal species during their own season).

References

1. Merkel B, Phillips RA, Descamps S, Yoccoz NG, Moe B, Strøm H. A probabilistic algorithm to process geolocation data. Mov Ecol [Internet]. 2016;4. Available from: http://dx.doi.org/10.1186/s40462-016-0091-8

2. Lazaridis E. lunar: Lunar Phase & Distance, Seasons and Other Environmental Factors (version 0.2-01). Available from CRAN. 2022.

3. Coleman M, Meier-Augenstein W. Ignoring IUPAC guidelines for measurement and reporting of stable isotope abundance values affects us all. Rapid Commun Mass Spectrom. 2014;28:1953–5.

4. McClintock BT, Michelot T. momentuHMM: R package for generalized hidden Markov models of animal movement. Methods Ecol Evol [Internet]. 2018;1–13. Available from: http://arxiv.org/abs/1710.03786%0Ahttp://dx.doi.org/10.1111/2041-210X.12995

5. Bivand RS, Pebesma EJ, Gómez-Rubio V, Pebesma EJ. Applied spatial data analysis with R. Springer; 2008.

6. Wessel P. Global distribution of seamounts inferred from gridded Geosat/ERS-1 altimetry. J Geophys Res Solid Earth [Internet]. 2001;106:19431–41. Available from: https://doi.org/10.1029/2000JB000083

7. Hijmans R, van Etten J, Cheng J, Mattiuzzi M, Sumner M, Greenberg JA, et al. Package ‘ raster ’. 2016; Available from: http://cran.r-project.org/package=raster

8. Hijmans RJ, Phillips S, Leathwick J, Elith J, Hijmans MRJ. Package ‘dismo.’ Circles. 2017;9:1–68.

9. Elith J, Leathwick JR, Hastie T. A working guide to boosted regression trees. J Anim Ecol. 2008;77:802–13.

10. Ventura F, Granadeiro JP, Padget O, Catry P. Gadfly petrels use knowledge of the windscape, not memorized foraging patches, to optimize foraging trips on ocean-wide scales. Proc R Soc B Biol Sci. 2020;287:20191775.
